# Enhanced structural and electrical properties of furnace-cooled SrZrO_3_-modified BFBT lead-free piezoceramics for high-temperature applications

**DOI:** 10.1038/s41598-025-24037-0

**Published:** 2025-11-17

**Authors:** Muhammad Kashif, Ali Ahmad Khan, Imran Hussain Khan, Muhammad Salman Habib, Muhammad Asif Rafiq, Adnan Maqbool, Fahim Ullah Jan, Saima Bibi, Nasimullah, Vojtech Blazek, Lukas Prokop

**Affiliations:** 1 Department of Metallurgical and Materials Engineering, University of Engineering and Technology (UET), Lahore, 54890 Pakistan; 2 Ionate Energy, London, UK; 3 Cecos University, Peshawar, Pakistan; 4 ENET Centre, VSB- Technichal University of Ostrava, Ostrava, Czech Republic

**Keywords:** Lead-free, Piezoceramics, SrZrO_3_-modified, BFBT-ceramics, Furnace-cooling, High-temperature, Dielectric, Microstructure, Refinement, Energy, Materials science, Physics

## Abstract

Lead-free SrZrO_3_ (SZ)-modified BiFeO_3_–BaTiO_3_ ceramics (BFBT–SZ*x*, *x* = 0, 0.02, and 0.04 mol%) were synthesized via a conventional solid-state reaction route followed by furnace cooling. X-ray diffraction (XRD) confirmed a single-phase pseudocubic perovskite structure with no secondary phases, while energy-dispersive X-ray spectroscopy (EDS) verified the presence of all intended elements, including Sr and Zr. Field emission scanning electron microscopy (FESEM) revealed enhanced grain uniformity and densification, with average powder particle size decreasing from 1.68 μm to 0.98 μm. The bulk absolute density increased from 3.35 g/cm^3^ (undoped) to 5.75 g/cm^3^ (SZ–0.04), corresponding to a relative density increase from ~ 90% (undoped) to ~ 92.5% (SZ–0.02) and ~ 94% (SZ–0.04). Fourier transform infrared spectroscopy (FTIR) confirmed the formation of metal–oxygen bonds characteristic of the perovskite lattice. Dielectric measurements showed improved thermal stability and reduced loss upon doping. The dielectric constant (*ε*_*r*_) decreased with increasing SZ content from ~ 7200 (*x* = 0) to ~ 2550 (*x* = 0.04) at 1 MHz, while the temperature of the *ε*_*r*_ peak shifted from ~ 350 ℃ (undoped) to > 400 ℃ (4% SZ) at 1 kHz. Doped samples maintained *ε*_*r*_ variation within ± 5% over a broad 20–510 °C range, and the dielectric loss (tan δ) was significantly reduced with low dielectric loss values on the order of 10^–3^ at 1 kHz, indicating diminished conduction losses. Increasing diffuseness parameter (*γ* = 1.69–1.89) confirmed relaxor behavior and enhanced polar disorder. Impedance spectroscopy revealed grain and grain boundary contributions in doped samples, while AC conductivity analysis indicated improved charge transport with temperature. Activation energy calculations showed a decrease from ~ 0.80 eV (*x* = 0) to ~ 0.20 eV (*x* = 0.04), confirming improved transport charge. The novelty of this work lies in demonstrating that low-level SZ doping simultaneously stabilizes the perovskite lattice, suppresses conduction loss, tunes relaxor-type response, and improves high-temperature dielectric reliability. These unique features make BFBT–SZ ceramics strong candidates not only for high-temperature dielectric and piezoelectric devices, as well as energy-related applications, including high-energy-density capacitors, energy storage systems, and thermally stable energy conversion modules.

## Introduction

The global interest in piezoelectric materials stems from their extensive use across various technologies, including photovoltaic systems, nano-devices, power transformers, sensing equipment, memory storage units, signal amplifiers, microwave filters, and phase modulators^1–7^. Currently, the piezoelectric market is predominantly led by lead-based ceramics, notably Lead Zirconate Titanate (PZT), owing to its outstanding ferroelectric and dielectric behaviors, straightforward processing, affordability, and high Curie temperature (~ 390 ℃)^8^. Within this class, the PbTiO_3_–PbZrO_3_ binary system has become a foundational material for use in sensors, actuators, and transducers^9^. However, these ceramics contain a significant amount of lead (exceeding 60%), and the emission of toxic PbO vapors during high-temperature sintering presents considerable health and environmental hazards^10^. As global environmental regulations become stricter, there is an urgent drive toward developing environmentally benign, lead-free alternatives^11,12^.

Among the promising multiferroic materials, bismuth ferrite (BiFeO_3_ or BFO) stands out due to its rhombohedral symmetry, single-phase crystallinity, and large spontaneous polarization (~ 100 µC/cm^2^)^13^. Nonetheless, BFO faces several processing challenges, such as thermal instability, secondary phase formation, and subpar resistive behavior. These drawbacks are mainly due to oxygen vacancy formation and partial reduction of Fe^3+^ to Fe^2+^ during sintering^4^. To address these shortcomings and improve the piezoelectric response, researchers have explored structural modification of ABO_3_-type perovskites via methods such as chemical etching, sol-gel synthesis, and ion substitution strategies^14^. Substitutions at the A-site, B-site, or both have proven effective in enhancing the properties of BFO-based ceramics^15^. The BiFeO_3_–BaTiO_3_ (BFBT) solid solution has gained attention for its improved ferroelectricity, piezoelectricity, and elevated Curie temperature^16^. However, one critical limitation of BFBT remains its high leakage current, often caused by bismuth loss during high-temperature treatment, given the volatile nature of Bi^17^. This challenge can be mitigated by introducing excess bismuth into the starting composition. Moreover, thermal processing—specifically furnace cooling—has shown potential in refining microstructural uniformity and stabilizing electrical performance of BFBT ceramics^18,19^.

Most of the recent research on lead-free piezoelectrics has focused on Na_0.5_Bi_0.5_TiO_3_ (NBT)-based systems due to their strong ferroelectric response and environmental safety. However, NBT suffers from a relatively low Curie temperature (~ 320 ℃) and limited high-temperature stability, which restricts its practical device applications. In contrast, BiFeO_3_–BaTiO_3_ (BFBT)-based multiferroics exhibit higher Curie temperatures (> 400 ℃), larger spontaneous polarization, and enhanced relaxor characteristics, making them more suitable for high-temperature dielectric and piezoelectric devices. Recent reports confirm that compositional modifications of BFBT systems can effectively reduce leakage current, improve thermal stability, and induce relaxor behavior, thereby addressing the intrinsic limitations of pure BiFeO_3_^20,21^. These findings further justify the present investigation on SrZrO_3_-modified BFBT ceramics.

Incorporating strontium zirconate (SrZrO_3_ or SZ), an ABO_3_-type perovskite with an orthorhombic crystal structure, into the BFBT matrix is anticipated to induce lattice distortion and bolster the electrical characteristics of the composite^22^. The leakage current is closely linked to the presence of oxygen vacancies. While substituting Sr^2+^ for Bi^3+^ introduces a charge imbalance that may not effectively suppress vacancy formation, replacing Fe^3+^ with Zr^4+^ maintains charge neutrality and can help mitigate oxygen deficiency, thus enhancing insulation performance^23^. The investigation of dielectric behavior and impedance spectroscopy is vital for understanding the electrical characteristics of both intra-grain and grain boundary regions, as well as their dependence on variables such as frequency and temperature^24^. To provide deeper insight into the electrical dynamics of this material system, a comprehensive analysis involving structural evaluation, dielectric response, and complex impedance behavior has been conducted.

The novelty of this research lies in the successful development of eco-friendly, lead-free BFBT–SZ*x*(*x* = 0, 0.02, and 0.04 mol%) piezoceramics synthesized through a conventional solid-state reaction method, followed by furnace cooling, a less energy-intensive, scalable alternative to quenching. This study systematically explores the influence of strontium zirconate doping on microstructural densification, dielectric relaxation, and electrical transport behavior, providing valuable insights into grain-boundary-controlled conduction and NTCR characteristics. The findings establish a foundation for cost-effective, sustainable alternatives in thermal sensing and dielectric applications.

Therefore, the present study aims to elucidate the influence of SrZrO_3_ incorporation on the structural, microstructural, dielectric, and impedance characteristics of BFBT ceramics synthesized via a conventional solid-state reaction method followed by furnace cooling. Emphasis is placed on understanding how Zr^4+^ substitution modulates oxygen vacancy concentration, electrical conductivity, and dielectric relaxation dynamics, thereby addressing the intrinsic leakage issues of BFO-based systems. Nonetheless, this investigation is confined to furnace-cooled BFBT–SZ*x*(*x* = 0, 0.02, and 0.04 mol%) compositions and does not encompass higher doping levels, alternative synthesis strategies, or comprehensive ferroelectric and piezoelectric characterizations, which remain as prospects for extending the scope of this work. This work aims to investigate the influence of SrZrO_3_ incorporation on the structure, dielectric relaxation, and conduction mechanisms of furnace-cooled BFBT ceramics. Unlike previous studies focusing on quenched or conventionally sintered systems, this work emphasizes furnace cooling as an eco-friendly and scalable route that yields improved dielectric stability and conductivity, making the ceramics highly suitable for high-temperature device applications.

## Materials and methods

**Fig. 1:**
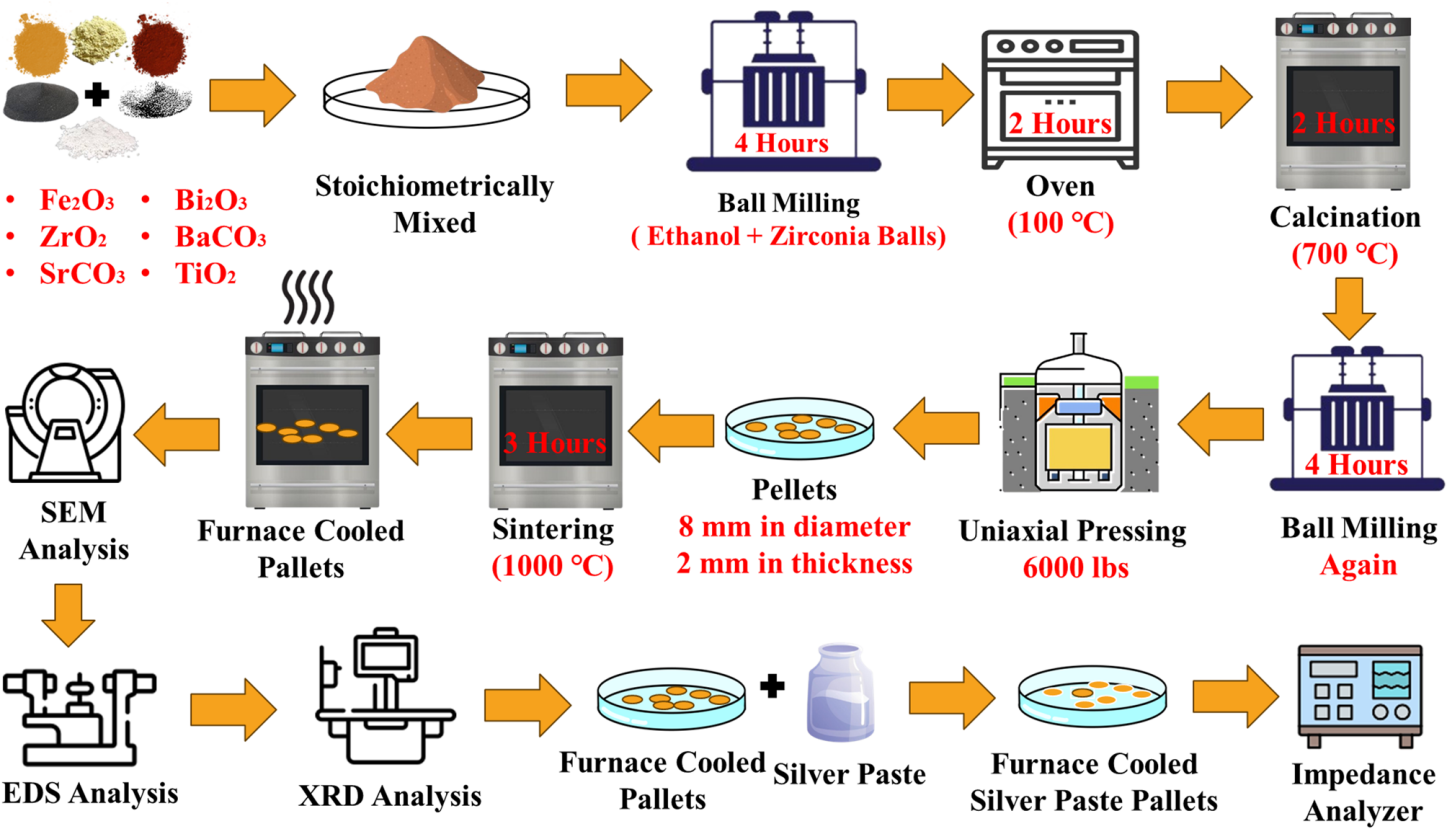
Schematic diagram of the solid-state synthesis route for BFBT–SZ ceramics. Stoichiometric raw powders were mixed and ball-milled in ethanol for 4 h, dried at 100 °C, and calcined at 700 °C for 2 h. The calcined powders were re-milled, pressed into pellets (8 mmx2 mm) under 6000 lbs, and sintered at 1000 °C for 3 h. The sintered samples were furnace-cooled, polished, silver-coated, and used for electrical characterization.

The (1–*x*)[(0.67Bi_1.05_FeO_3_ – 0.33BaTiO_3_)] – *x*SrZrO_3_ (BFBT–SZ*x*), where *x* = 0, 0.02, and 0.04 mol%, ceramic compositions were synthesized using the conventional solid-state reaction route as illustrated in Fig. 1. High-purity analytical-grade precursors—Bi_2_O_3_ (Daejung Chemicals and Metals Co. Ltd.), Fe_2_O_3_ (UNI-CHEM), BaCO_3_ (Riedel-de-Haën), TiO_2_ (AnalaR), ZrO_2_ (UNI-CHEM), and SrCO_3_ (International Lab, USA)—were weighed in stoichiometric proportions. The raw materials were thoroughly mixed and milled in ethanol using zirconia balls for 4 h to ensure homogeneity. The slurry was then dried at 100 ℃ for 2 h, followed by calcination at 700 ℃ for 2 h to initiate solid-state reactions. The overall chemical reaction during synthesis can be represented as:

(1–*x*)[0.67Bi_2_O_3_ + 1.05Fe_2_O_3_ + 0.33BaCO_3_ + 0.33TiO_2_] + *x*[SrCO_3_ + ZrO_2_] → (1–*x*)[(0.67Bi_1.05_FeO_3_–0.33BaTiO_3_)] – *x*SrZrO_3_ + gaseous by-products (CO_2_).

Here, the decomposition of BaCO_3_ and SrCO_3_ releases CO_2_, and the resulting oxides react to form the desired perovskite solid solution. After calcination, the powder was re-milled for another 4 h to further improve uniformity. The homogenized powder was compacted into disc-shaped pellets (8 mm diameter, 2 mm thickness) under a uniaxial pressure of 6000 lbs. These pellets were subsequently sintered in ambient air at 1000 ℃ for 3 h in a box furnace. Instead of rapid cooling or quenching, the samples were allowed to cool slowly within the furnace to room temperature under natural thermal relaxation conditions to stabilize the microstructure and reduce thermal stresses.

Phase identification of the sintered ceramics was performed using X-ray diffraction (XRD, Bruker D8 Advance, Cu Kα radiation, λ = 1.5406 Å, 2θ = 20–80°, step size = 0.02°, scan rate = 1°/min). The microstructure was examined by Field Emission Scanning Electron Microscopy (FESEM, JEOL JSM-7600 F, accelerating voltage 15 kV, working distance 10 mm), and elemental analysis was carried out using Energy Dispersive X-ray Spectroscopy (EDS, Oxford Instruments X-Max, 20 mm² detector, resolution 127 eV at Mn Kα) attached to the FESEM. Functional group identification was performed for the powder sample using Fourier Transform Infrared Spectroscopy (FTIR, Bruker Tensor 27, spectral range 4000–500 cm^− 1^, resolution 4 cm^− 1^, 32 scans per spectrum). For electrical characterization, the sintered pellets were polished and coated with silver paste as electrodes. Impedance spectroscopy (IS) was performed using a HIOKI IM3536 LCR Meter in the frequency range of 100 Hz – 1 MHz and temperature range of 300–500 ℃. Measurements were carried out in a programmable furnace with a controlled cooling rate of 25 ℃/min to ensure a reliable thermal response.

## Results and discussion

### Structural studies

**Fig. 2:**
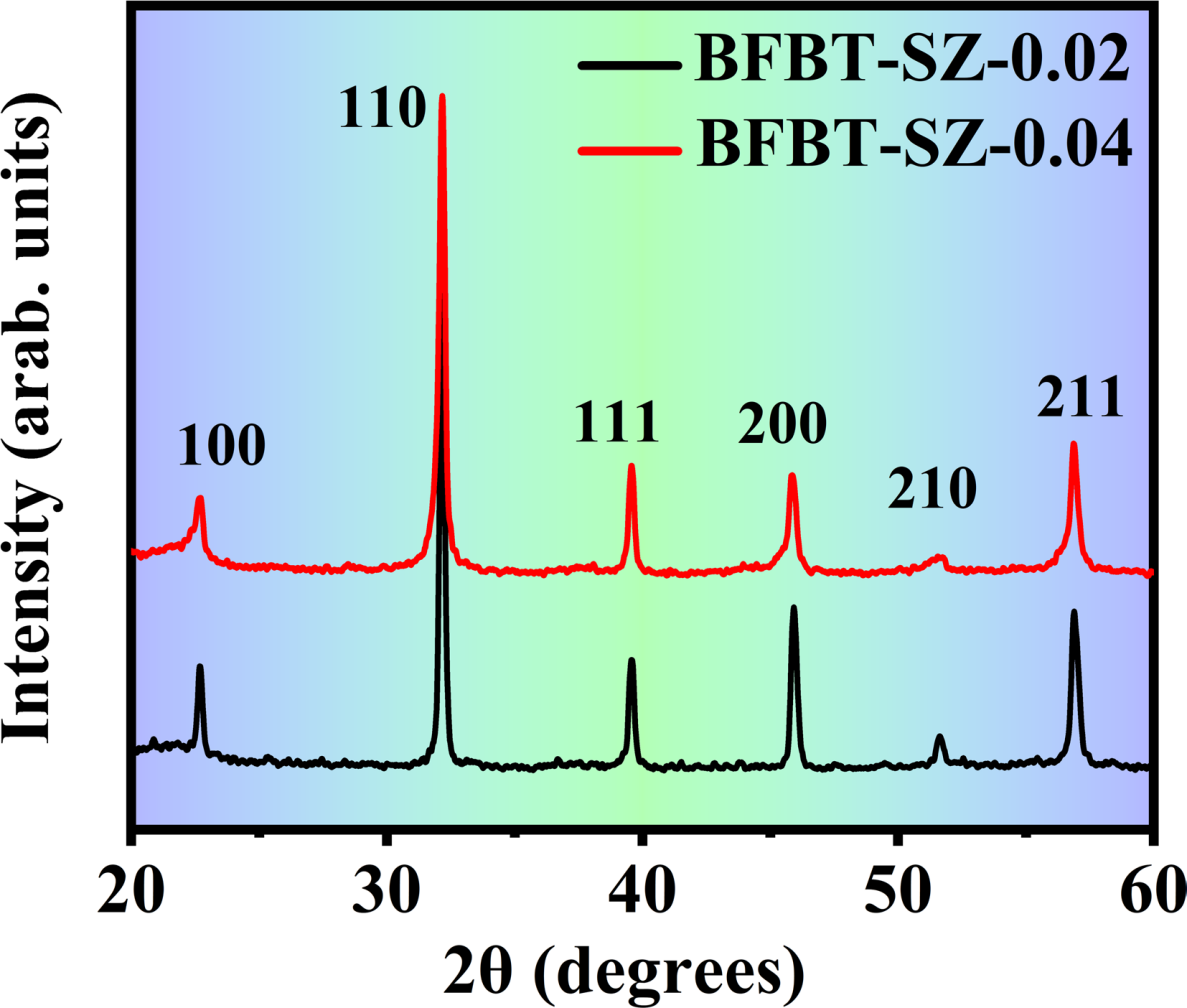
XRD patterns of furnace-cooled BFBT–SZ*x* ceramics (*x* = 0.02 and 0.04 mol%), showing pseudocubic perovskite phase without detectable secondary phases.

X-ray diffraction (XRD) was employed to examine the phase purity and crystallographic structure of furnace-cooled BFBT–SZ*x*(*x* = 0.02 and 0.04 mol%) ceramics. The XRD patterns, as shown in Fig. 2, confirm the formation of a single-phase perovskite structure with pseudocubic symmetry. The major reflections correspond to the (100), (110), (111), (200), (210), and (211) planes, typical of perovskite-type structures. No secondary phases or impurity peaks were observed within the detection limit of the instrument, indicating that SrZrO_3_ was successfully incorporated into the BFBT matrix without phase decomposition or structural separation^25^. Importantly, the samples were cooled slowly within the furnace, allowing for equilibrium phase stabilization and the suppression of unwanted structural transformations. The sharp and well-defined peaks suggest good crystallinity, and the consistent peak positions across both doping levels (*x* = 0.02 and 0.04 mol%) further confirm structural homogeneity.

Although the undoped BFBT (*x* = 0) pattern is not presented here, its perovskite structure and pseudocubic (or rhombohedral-to-pseudocubic) symmetry have been well established in the literature^27,75^, which supports the present findings. In the current study, the lattice parameters and phase purity were evaluated through indexing and least-squares fitting of the observed reflections, confirming the pseudocubic crystalline structure with adequate precision for the intended scope. Similar methodologies have also been reported in earlier investigations on BF–BT systems^28^. While the present analysis based on indexing and least-squares fitting adequately confirms the pseudocubic crystalline structure, advanced refinements such as Rietveld analysis will be valuable in future studies to obtain precise lattice parameters and quantitative phase information. Overall, the results obtained here are in strong agreement with previously reported lead-free perovskite systems^27,28^, further validating the successful synthesis of dense, single-phase BFBT–SZ ceramics through furnace-cooled processing. These comparisons confirm that the observed diffraction peaks correspond to the expected pseudocubic perovskite phase, thereby validating the successful synthesis of dense, single-phase BFBT–SZ ceramics through furnace-cooled processing^27,28^.

### Powder particle size and density analysis

**Fig. 3:**
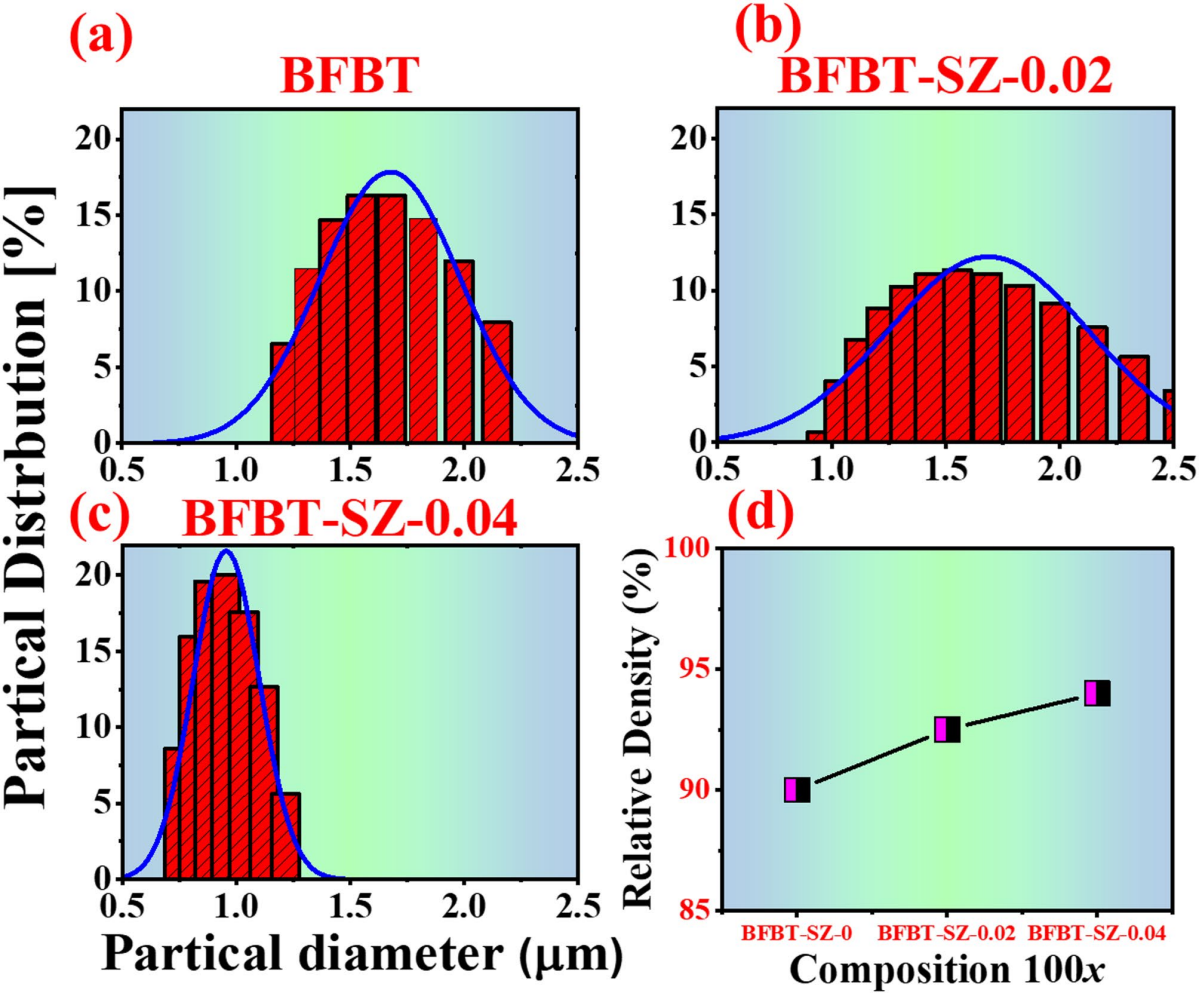
**(a–c)** Particle size distribution of BFBT–SZ*x* ceramics (*x* = 0, 0.02, and 0.04 mol%), **(d)** Relative density values showing increased densification with SZ doping.

Powder particle size analysis was performed using a BM10 Litesizer 500 particle analyzer. For each composition, representative powder samples were dispersed into a suitable medium and analyzed to determine the particle size distribution. The resulting histograms for undoped and SrZrO_3_ (SZ)-doped compositions are shown in Fig. 3a–c. The average particle diameter of the undoped BFBT powder was approximately 1.68 μm. Upon doping with 0.02 mol% SZ, the particle size decreased to 1.55 μm, and a further reduction to 0.98 μm was recorded for the 0.04 mol% SZ-doped sample. This progressive reduction in particle size with increasing SZ content is attributed to the suppression of grain coarsening, likely due to the incorporation of larger Sr^2+^ and Zr^4+^ ions that inhibit grain boundary mobility during synthesis and calcination processes^29^.

Figure 3d presents the relative density values of the BFBT–SZ*x* ceramics (x = 0, 0.02, and 0.04 mol%). A clear trend of increasing density is observed with rising SZ content. The undoped BFBT sample exhibits the lowest absolute bulk density of 3.35 g/cm^3^, corresponding to a relative density of ~ 90%. In contrast, the SZ–0.02 and SZ–0.04 samples show higher relative densities of ~ 92.5% and ~ 94%, respectively, with the BFBT–SZ–0.04 composition reaching a maximum bulk absolute density of 5.75 g/cm^3^.

The relative density (RD) of the sintered samples was calculated using the relation^30^:

$$\:\text{RD}{\%}\text{}\text{=}\text{}\frac{{{\rho}}_{\text{bulk}}}{{{\rho}}_{\text{theoretical}}}{\times 100}$$

Where $\:{{\rho}}_{\text{bulk}}$ is the experimentally measured bulk density (Archimedes’ method) and $\:{{\rho}}_{\text{theoretical}}$​ is the theoretical density obtained from Rietveld refinement of the XRD data^31^. These results clearly demonstrate that SrZrO_3_ doping enhances densification in BFBT ceramics, as the relative density increases with dopant concentration^32^. The enhancement in densification is attributed to the SZ-induced inhibition of grain growth and improved particle packing efficiency. The presence of larger dopant ions modifies the sintering dynamics, promoting a more compact microstructure with reduced porosity. These results demonstrate that SZ doping enhances densification, which is expected to improve structural integrity and potentially boost the electrical performance of the ceramics^29^.

### Microstructural analysis (FESEM)

**Fig. 4:**
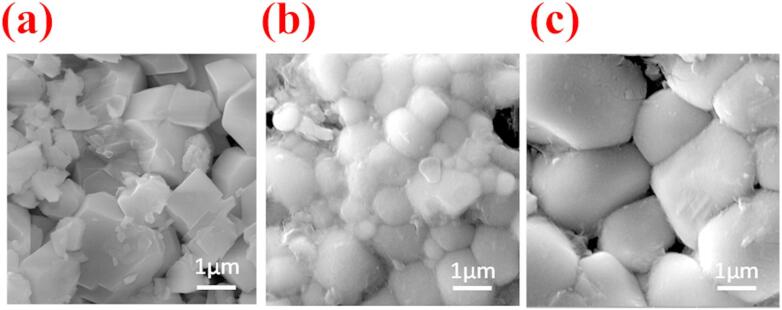
FESEM micrographs of furnace-cooled BFBT–SZ*x* ceramics: **(a)** FC–BFBT–SZ–0, **(b)** FC–BFBT–SZ–0.02, and **(c)** FC–BFBT–SZ–0.04, showing enhanced grain densification and connectivity with increasing SZ content.

Field Emission Scanning Electron Microscopy (FESEM) was employed to investigate the surface morphology and grain structure of furnace-cooled FC–BFBT–SZ*x* ceramics with *x* = 0, 0.02, and 0.04 mol%, as illustrated in Fig. 4. The undoped BFBT sample (FC–BFBT–SZ–0) exhibits irregular, coarse grains with significant porosity and poor grain-to-grain connectivity, indicative of incomplete densification during sintering. Upon incorporation of 0.02 mol% SrZrO_3_ (FC–BFBT–SZ–0.02), the microstructure shows a notable refinement, with smaller and more uniformly distributed grains. The improved grain packing and reduced pore volume suggest enhanced densification, likely facilitated by the partial substitution of SrZrO_3_, which modifies sintering behavior and promotes grain boundary diffusion. The improvement in densification and grain refinement with SrZrO_3_ addition can be ascribed to dopant-induced modifications of sintering kinetics: the substitution of Sr^2+^ and Zr^4+^ ions introduces lattice strain and defect states (e.g., oxygen vacancies), which accelerate mass transport along grain boundaries. This enhanced grain boundary diffusion not only aids in pore elimination but also suppresses abnormal grain growth, thereby promoting controlled coalescence and homogeneous grain development.

The FC–BFBT–SZ–0.04 sample reveals a further enhancement in microstructural features. Grains appear well-developed with smoother surfaces and distinct boundaries, while intergranular voids are significantly minimized. This indicates that higher SZ doping concentrations (0.04 mol%) effectively suppress abnormal grain growth and promote homogeneous grain coalescence, leading to a more compact and densified microstructure. These microstructural trends are consistent with the earlier particle size and density measurements, wherein the FC–BFBT–SZ–0.04 composition exhibited the finest average particle size and the highest relative density. The reduced porosity and refined grains observed in FESEM directly contribute to the improved dielectric performance of the ceramics. Enhanced densification lowers leakage paths and defect concentrations, leading to reduced dielectric loss and more stable polarization, while uniform grain connectivity facilitates efficient charge transport across the microstructure. These features correlate well with the enhanced thermal stability and relaxor-type behavior observed in the dielectric measurements^33^.

### Energy dispersive X-ray spectroscopy (EDS)

**Fig. 5:**
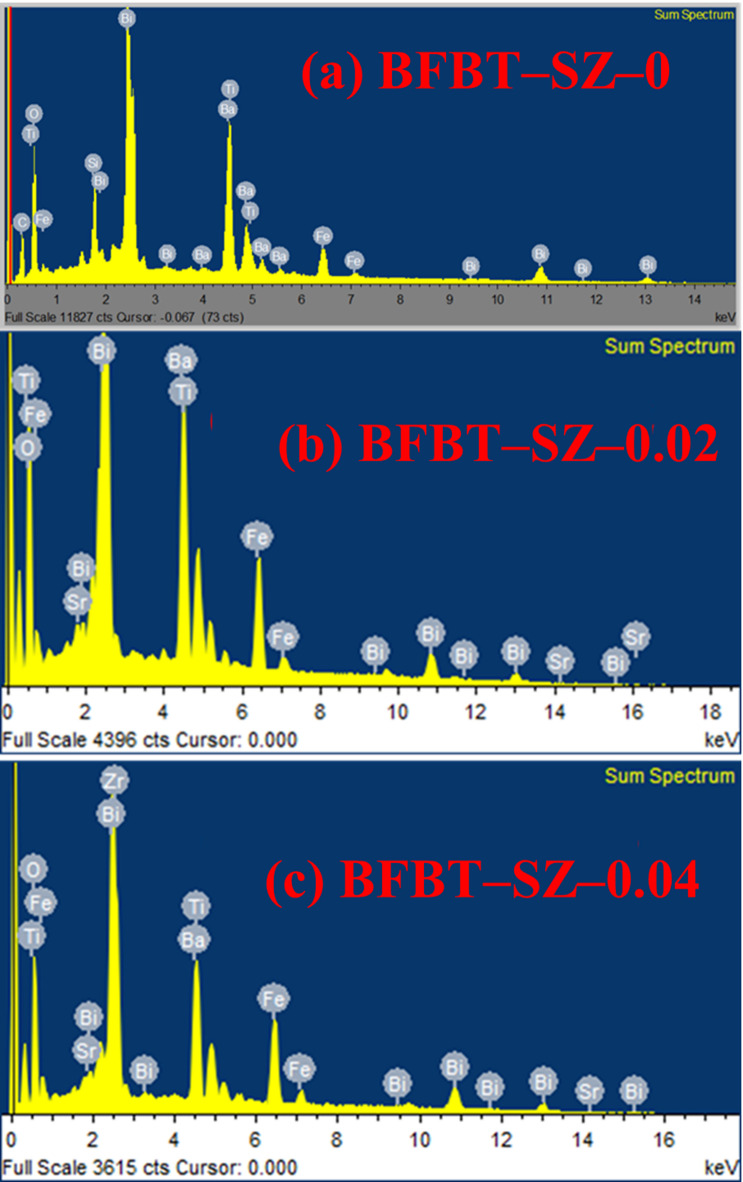
**(a)** EDS spectrum of undoped BFBT piezoceramics showing the presence of Ba, Bi, Fe, Ti, and O. Minor peaks corresponding to Si and C originate from the sample holder and carbon coating, respectively, confirming the elemental composition and purity of the synthesized material. **(b-c)** EDS spectra of BFBT–SZ*x* ceramics (*x* = 0.02 and 0.04 mol%) display additional Sr and Zr peaks alongside the primary BFBT elements. The detection of all intended elements without extraneous signals confirms high material purity and effective incorporation of SrZrO_3_ dopants.

Energy-dispersive X-ray spectroscopy (EDS) was conducted to analyze the elemental composition of the synthesized furnace-cooled ceramics. Figure 5a displays the EDS spectrum of the undoped BFBT piezoceramic, confirming the presence of the expected elements: barium (Ba), bismuth (Bi), iron (Fe), titanium (Ti), and oxygen (O). Minor peaks corresponding to silicon (Si) and carbon (C) are also observed, which are attributed to the sample holder and the conductive carbon coating used during measurement. The detection of all principal elements without foreign contaminants confirms the successful synthesis of high-purity, lead-free BFBT ceramics.

In the doped samples (FC–BFBT–SZ), the EDS spectra shown in Fig. 5b–c reveal additional peaks corresponding to strontium (Sr) and zirconium (Zr), along with the original BFBT constituents, confirming the effective incorporation of SrZrO_3_ into the lattice. However, while EDS reliably identifies major constituent elements, it has inherent limitations: its sensitivity to light elements (such as O) is relatively poor, and it cannot fully resolve trace impurities or detect subtle secondary phases present in low concentrations. For this reason, EDS alone cannot guarantee crystallographic phase purity. To overcome this limitation, XRD analysis was employed in parallel, which provides information on crystal structure and phase composition. The combined use of EDS and XRD thus ensures both elemental verification and confirmation of the single-phase perovskite structure, ruling out the presence of impurity phases^34^.

Semi-quantitative EDS analysis was also performed to estimate the elemental composition of the undoped and SrZrO_3_-doped BFBT ceramics. The calculated atomic % and weight% values are summarized in Table 1. All expected constituent elements (Bi, Fe, Ba, Ti, and O) were detected in the parent BFBT sample, with additional Sr and Zr peaks appearing in the doped compositions, confirming the incorporation of SrZrO_3_. It should be noted that the oxygen content appears underestimated due to the limited sensitivity of EDS for light elements, which is a well-documented limitation^35–37^. Nonetheless, the overall cation ratios are in good agreement with the nominal stoichiometry.

**Table 1:** Semi-quantitative elemental composition (atomic % and weight%) of undoped and SrZrO_3_-doped BFBT ceramics obtained from EDS analysis.

Element	FC–BFBT–SZ–0		FC–BFBT–SZ–0.02		FC–BFBT–SZ–0.04	
	Atomic %	Weight%	Atomic %	Weight%	Atomic %	Weight%
Bi	15.6	41.2	15.2	40.5	15.0	39.8
Fe	14.8	18.9	14.5	18.7	14.2	18.2
Ba	7.9	20.3	7.6	19.6	7.4	19.2
Ti	7.6	8.1	7.3	7.9	7.0	7.6
Sr	–	–	1.1	1.6	2.2	3.1
Zr	–	–	0.9	1.3	1.8	2.6
O	54.1	11.5	53.4	10.4	52.4	9.5

### FTIR spectroscopic characterization

**Fig. 6:**
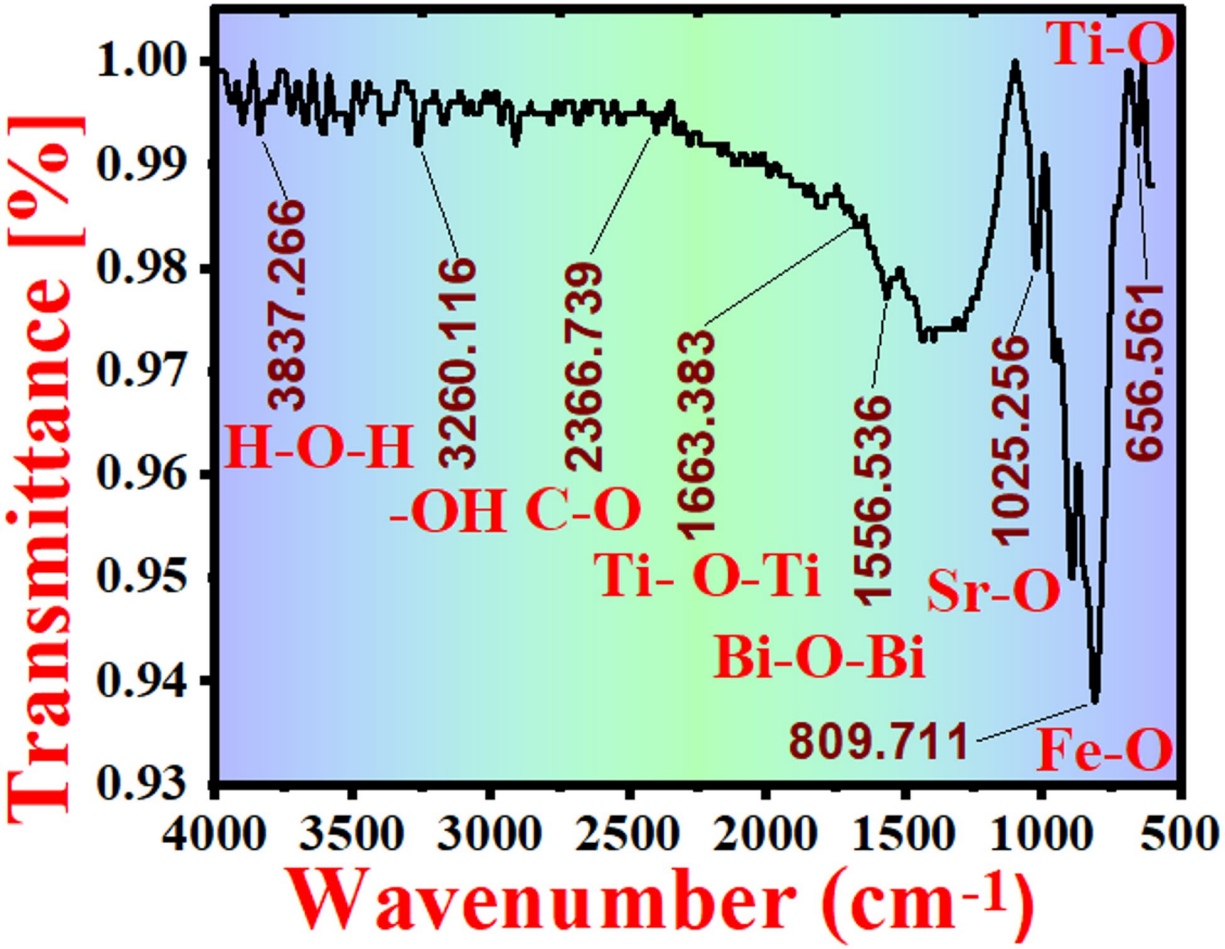
FTIR spectrum of the BFBT–SZ system in the range of 4000–500 cm^−1^, indicating the presence of hydroxyl groups, adsorbed water, and metal–oxygen (Ti–O, Bi–O, Sr–O, Fe–O) bonding vibrations characteristic of the perovskite structure.

In this study, FTIR analysis was performed only for the powder sample of the BFBT–SZ composition to qualitatively confirm the functional groups and metal–oxygen vibrations, rather than for all compositions. Fourier Transform Infrared (FTIR) spectroscopy was performed in the range of 4000–500 cm^− 1^ to identify the functional groups and metal–oxygen bonding environments in the powder-doped sample of BFBT–SZ ceramic system, as shown in Fig. 6. The broad absorption bands observed at approximately 3837 cm^− 1^ and 3260 cm^− 1^ are assigned to the stretching vibrations of H–O–H and hydroxyl (–OH) groups, respectively, indicating the presence of physisorbed water and surface hydroxylation. A weak band around 2366 cm^− 1^ corresponds to C–O stretching vibrations, likely due to ambient CO₂ adsorption on the sample surface.

Distinct bands at 1663 cm^− 1^ and 1556 cm^− 1^ are attributed to Ti–O–Ti and Bi–O–Bi vibrations, confirming the formation of titanium and bismuth oxide substructures within the perovskite matrix. Moreover, absorption peaks at 1025 cm^− 1^, 809 cm^− 1^, and 656 cm^− 1^ are associated with Sr–O, Ti–O, and Fe–O bond vibrations, respectively. These observations indicate the coexistence of various metal–oxygen environments that are consistent with the designed BFBT–SZ ceramic system.

It should be noted that FTIR provides qualitative information about vibrational modes and bonding but does not directly confirm the lattice incorporation of Sr and Zr dopants. Therefore, while the presence of Sr–O and related vibrations supports the expected bonding environment, the successful incorporation of Sr and Zr into the BFBT lattice is more reliably validated through complementary structural and compositional analyses such as XRD and EDS. Overall, the FTIR results corroborate the expected chemical framework of the BFBT–SZ ceramics and are in good agreement with the findings from XRD and EDS analyses^38^.

### Dielectric study as a function of temperature.

**Fig. 7:**
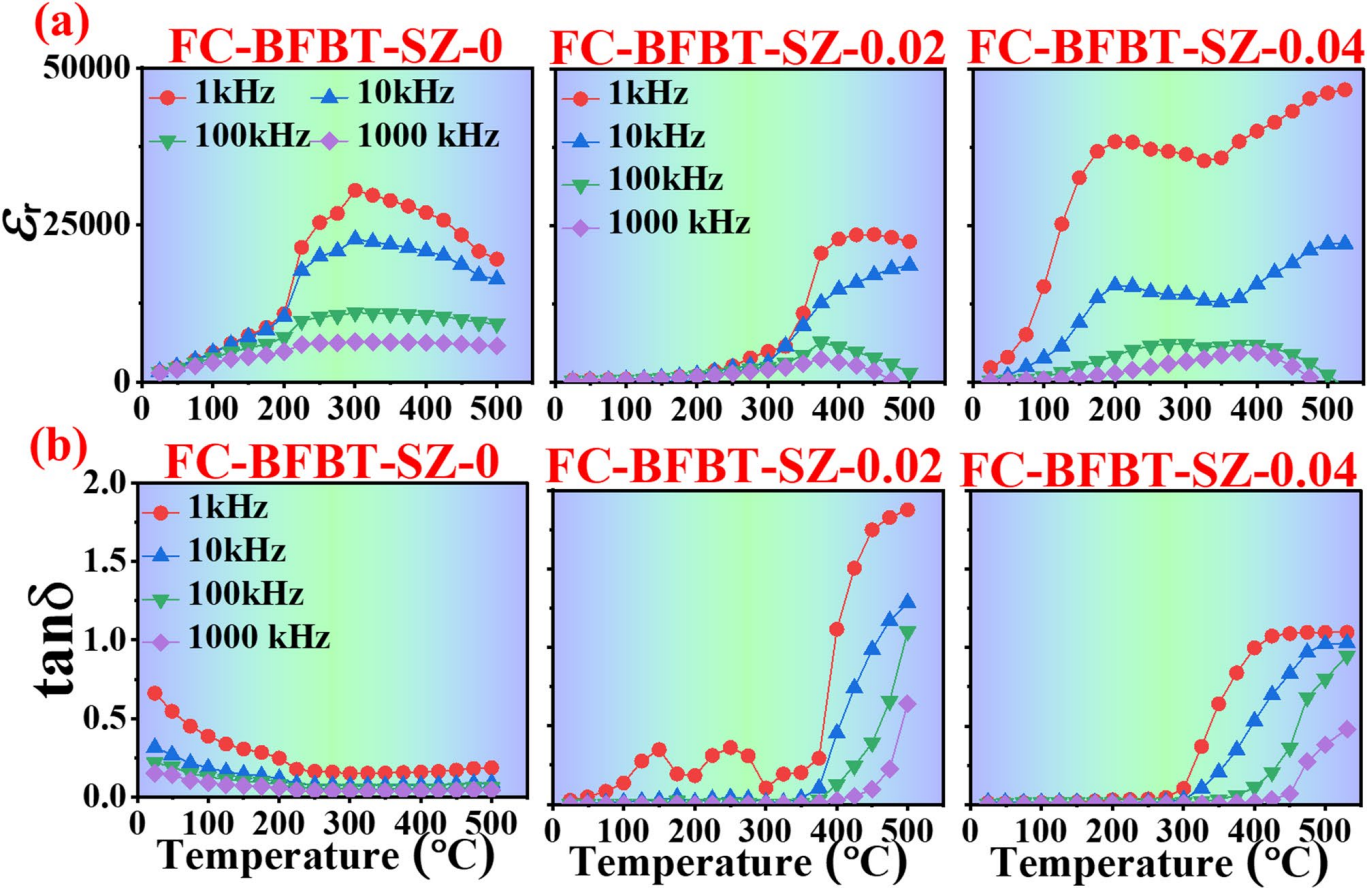
Temperature dependence of **(a)** dielectric constant (*ε*_*r*_) and **(b)** dielectric loss (tan δ) for FC–BFBT–SZ*x* ceramics (*x* = 0, 0.02, 0.04 mol%). The dielectric peak shifts to higher temperatures with SZ doping, indicating better thermal stability, while dielectric loss rises near the transition and decreases with frequency.

The dielectric constant (*ε*_*r*_) and dielectric loss (tan δ) of furnace-cooled FC–BFBT–SZ*x* ceramics (*x* = 0, 0.02, and 0.04 mol%) were investigated as functions of temperature and frequency, as illustrated in Fig. 7. The dielectric constant demonstrates a temperature-dependent increase, peaking at the ferroelectric–paraelectric phase transition before declining at higher temperatures—behavior typical of ferroelectric materials^39,40^. Both the magnitude and temperature position of the *ε*_*r*_ peak are influenced by the SZ doping concentration and measurement frequency. At 1 kHz, the dielectric constant reaches its maximum due to enhanced dipolar polarization. However, as the frequency increases to 10 kHz, 100 kHz, and 1 MHz, *ε*_*r*_ progressively decreases, as dipoles are unable to realign with the increasingly rapid alternating electric field. In the undoped (FC–BFBT–SZ–0) composition, a broad *ε*_*r*_ peak appears between 300 ℃ and 400 ℃, accompanied by a steep decline at higher frequencies. With 2 mol% SZ doping (FC–BFBT–SZ–0.02), the peak shifts slightly toward higher temperatures, indicating a stabilization of the ferroelectric phase and an expanded temperature range for elevated dielectric performance. At 4 mol% doping (FC-BFBT–SZ–0.04), the peak shifts even further, suggesting enhanced thermal stability and a more delayed ferroelectric-to-paraelectric transition.

The dielectric loss (tan δ) profiles complement these observations. At low temperatures, all samples exhibit minimal tan δ values, indicating low energy dissipation and negligible conduction losses. As temperature increases and approaches the ferroelectric transition, tan δ rises sharply due to increased charge carrier mobility and leakage conduction. This rise is more pronounced at lower frequencies, where carriers are more capable of responding to the applied field. At higher frequencies, tan δ values decrease, as carriers cannot follow the rapid oscillations effectively. Specifically, the undoped (FC–BFBT–SZ–0) sample shows a steady increase in tan δ, peaking between 300 ℃ and 400 ℃. For FC–BFBT–SZ–0.02, the peak is delayed and slightly more elevated, reflecting improved dielectric phase stability. In the case of FC-BFBT–SZ–0.04, the loss peak shifts further to higher temperatures, and tan δ values remain lower at low temperatures, signifying improved energy efficiency and reduced conduction losses.

Overall, SZ doping improves the dielectric performance of FC–BFBT ceramics by enhancing thermal stability, increasing the transition temperature, and extending the temperature range over which a high dielectric constant is maintained. Additionally, higher SZ concentrations reduce dielectric loss at lower temperatures, making the material more efficient. The strong frequency dependence further confirms that dielectric behavior is governed by dipolar polarization and conduction mechanisms that are sensitive to both dopant concentration and field frequency. These enhancements render SZ-doped FC–BFBT ceramics promising candidates for high-temperature capacitor and electronic device applications^41^.

**Fig. 8:**
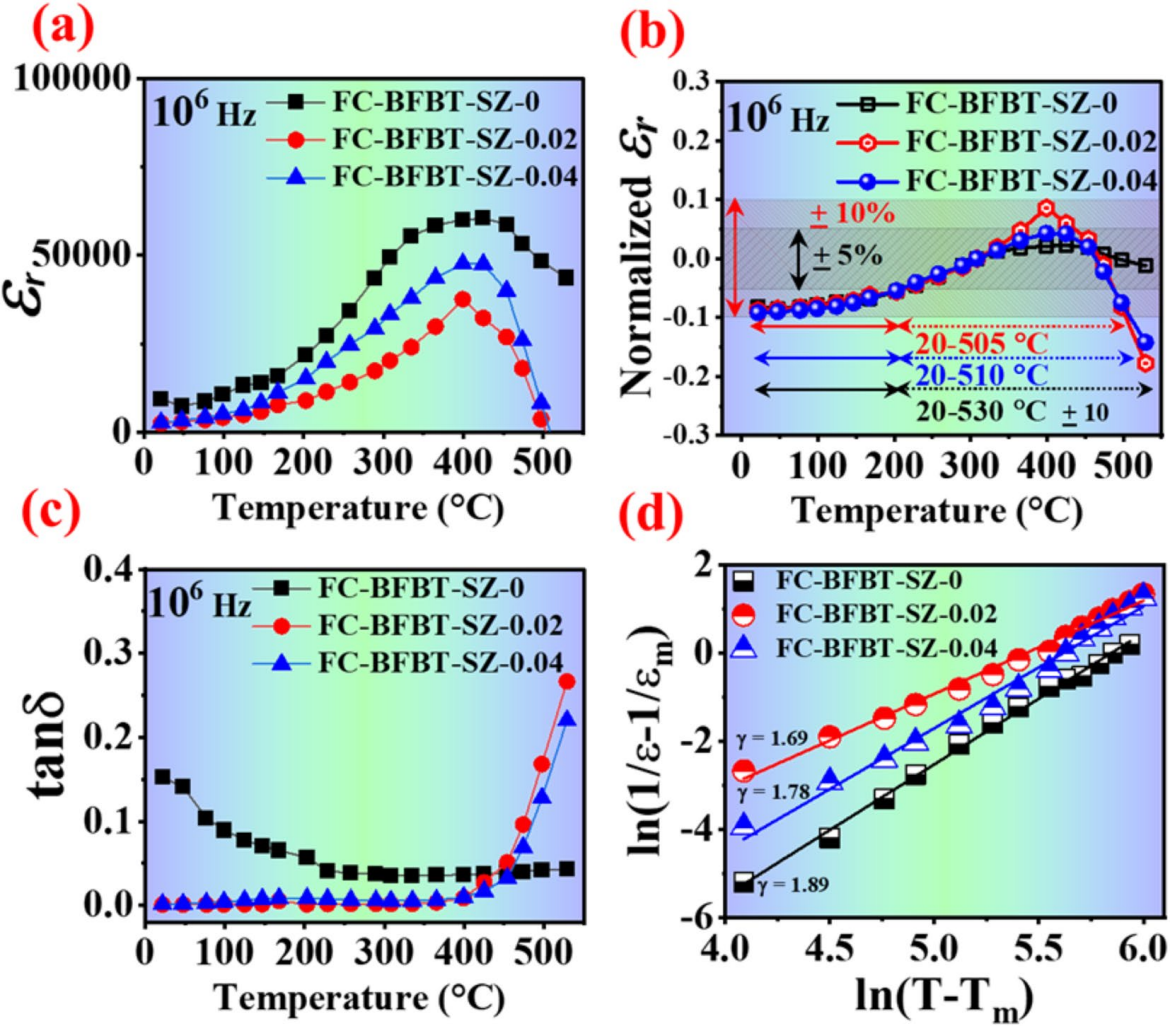
Dielectric and electrical properties of furnace-cooled FC–BFBT–SZ*x* ceramics (*x* = 0, 0.02, and 0.04 mol%) at 1 MHz. **(a)** dielectric constant decreases with doping, **(b)** normalized dielectric constant shows better thermal stability, **(c)** dielectric loss is reduced, and **(d)** Curie–Weiss analysis indicates relaxor behavior with higher *γ* values.

Figure 8a–d illustrates the dielectric and electrical properties of furnace-cooled FC–BFBT–SZ*x* ceramics *(x* = 0, 0.02, and 0.04 mol%) at a frequency of 1 MHz. As shown in part (a), the dielectric constant (*ε*_*r*_) increases with temperature and reaches a maximum (*ε*_*m*_​) near the transition temperature (*T*_*m*_​). The undoped sample (FC-BFBT–SZ-0) exhibits the highest peak dielectric constant (~ 7200), followed by FC-BFBT–SZ–0.02 and FC-BFBT–SZ–0.04, indicating a decline in dielectric response with increased SrZrO_3_ content. This trend suggests that SZ doping suppresses space charge polarization, reduces defect density, and promotes a more stable microstructure with lower dielectric activity^42,43^. Furthermore, the broadening and reduction in *ε*_*r*_ with doping are indicative of diffuse phase transitions and relaxor behavior, consistent with the disruption of long-range ferroelectric ordering, as commonly observed in perovskite-based relaxors^44^. In Fig. 8b, the normalized dielectric constant demonstrates that the doped compositions, especially FC-BFBT–SZ–0.02 and FC-BFBT–SZ–0.04, exhibit enhanced thermal stability by maintaining variation within ± 5% across a wide temperature range (20–510°C), a crucial factor for device reliability^45^. Dielectric loss (tan δ) data in part 8c reveal that SZ doping substantially lowers tan δ from 100 to 450 ℃, which may be attributed to reduced leakage current and suppressed hopping conduction of oxygen vacancies^46^. In part 8d, the relaxor behavior is analyzed using the modified Curie–Weiss law (1/*ε*_*r*_ − 1/*ε*_*m*_ = (*T* − *T*_*m*_)^γ^ / C) where *ε*_*r*_, *ε*_*m*_, *γ*, and C represent the dielectric constant, maximum dielectric constant, relaxor coefficient, and Curie–Weiss constant, respectively. The linear relationship between ln(*T* − *T*_*m*_​) and ln(1/*ε*_*r*_ − 1/*ε*_*m*_) yields increasing values of the relaxation exponent *γ*:1.69 for SZ–0, 1.78 for SZ–0.02, and 1.89 for SZ–0.04^47^. These *γ* values (*γ* > 1) confirm the transition from normal ferroelectric to strong relaxor ferroelectric behavior with doping, likely due to the formation of polar nanoregions and increased compositional disorder^44,48^. This progressive shift is consistent with reported behaviors in BiFeO_3_-based systems and supports the development of thermally stable, multifunctional piezoceramics.

Overall, while the suppression of space charge polarization and defect-mediated contributions reduces *ε*_*m*_, this trade-off provides significant benefits in terms of dielectric stability, reduced loss, and improved thermal reliability. Such a balance is particularly advantageous for high-temperature capacitors and electronic applications, where stable performance and efficiency are prioritized over achieving the highest possible permittivity values.

### Comparison of dielectric properties with literature

As shown in Table 2, SZ doping in FC–BFBT ceramics significantly improves dielectric performance by enhancing thermal stability, reducing dielectric loss, and inducing relaxor behavior. While the undoped sample shows a high *ε*_*r*_ (~ 7200), it suffers from high losses and poor stability. Doped compositions (SZ–0.02 and SZ–0.04) exhibit lower *ε*_*r*_ but broader phase transitions, stable dielectric response (± 5% from 20 to 510°C), and stronger relaxor behavior (*γ* up to 1.89). Compared to literature-reported La-, Nd-, and SrZrO_3_-doped BiFeO_3_–BaTiO_3_ systems, the present SZ-doped ceramics show comparable or better relaxor characteristics and lower energy dissipation, making them suitable for high-temperature capacitor applications.

**Table 2:** Comparison of dielectric properties of FC–BFBT–SZ*x* ceramics (*x* = 0, 0.02, and 0.04 mol%) with related BiFeO_3_–BaTiO_3_-based systems from literature. The table summarizes dielectric constant (*ε*_*r*_), dielectric loss (tan δ), transition temperature shift, thermal stability, and relaxor behavior (*γ*) to highlight the effect of SrZrO_3_ doping on the dielectric performance and thermal reliability of BFBT ceramics.

Material System	ε_*r*_ (1 MHz, ~Tm)	tan δ (100–450 °C)	T_m_ Shift with Doping	Thermal Stability (± 5%) Range	Relaxor Behavior (γ)	Key Features	References
FC–BFBT–SZ–0	~ 7200	High	—	Narrow	1.69	High *ε*_*r*_, low stability	Present Study
FC–BFBT–SZ–02	~ 2883	Moderate	Slight ↑	20–510 °C	1.78	Improved stability, reduced tan δ	Present Study
FC–BFBT–SZ–04	~ 2550	Low	Further ↑	20–510 °C	1.89	Best thermal & dielectric efficiency	Present Study
BiFeO_3_–BaTiO_3_ (undoped)	~ 6000	High	—	Narrow	1.9	High *ε*_*r*_, poor loss control	^49,50^
BiFeO_3_–BaTiO_3_–La	~ 3500	Moderate	Moderate ↑	Broad	2.5	Stable relaxor behavior	^51,52^
BiFeO_3_–BaTiO_3_–SrZrO_3_	~ 2800	Low	Significant ↑	Broad	2.8	Enhanced relaxor, low loss	^50,53^
BiFeO₃–BaTiO₃–Nd	~ 3000	Low	Slight ↑	Moderate	2.6	Good relaxor with low conduction	^54,55^

### Complex impedance spectroscopy (CIS) and electrical properties

Impedance spectroscopy (IS) is a powerful technique for investigating the electrical response of materials and the contribution of their microstructural features. In multiferroic systems, impedance measurements are typically conducted over a frequency range of 100 Hz to 1 MHz and a temperature range of 300–500 ℃^56^.

### Nyquist (cole–cole) plot of furnace-cooled (FC–BFBT–SZ) ceramics

**Fig. 9:**
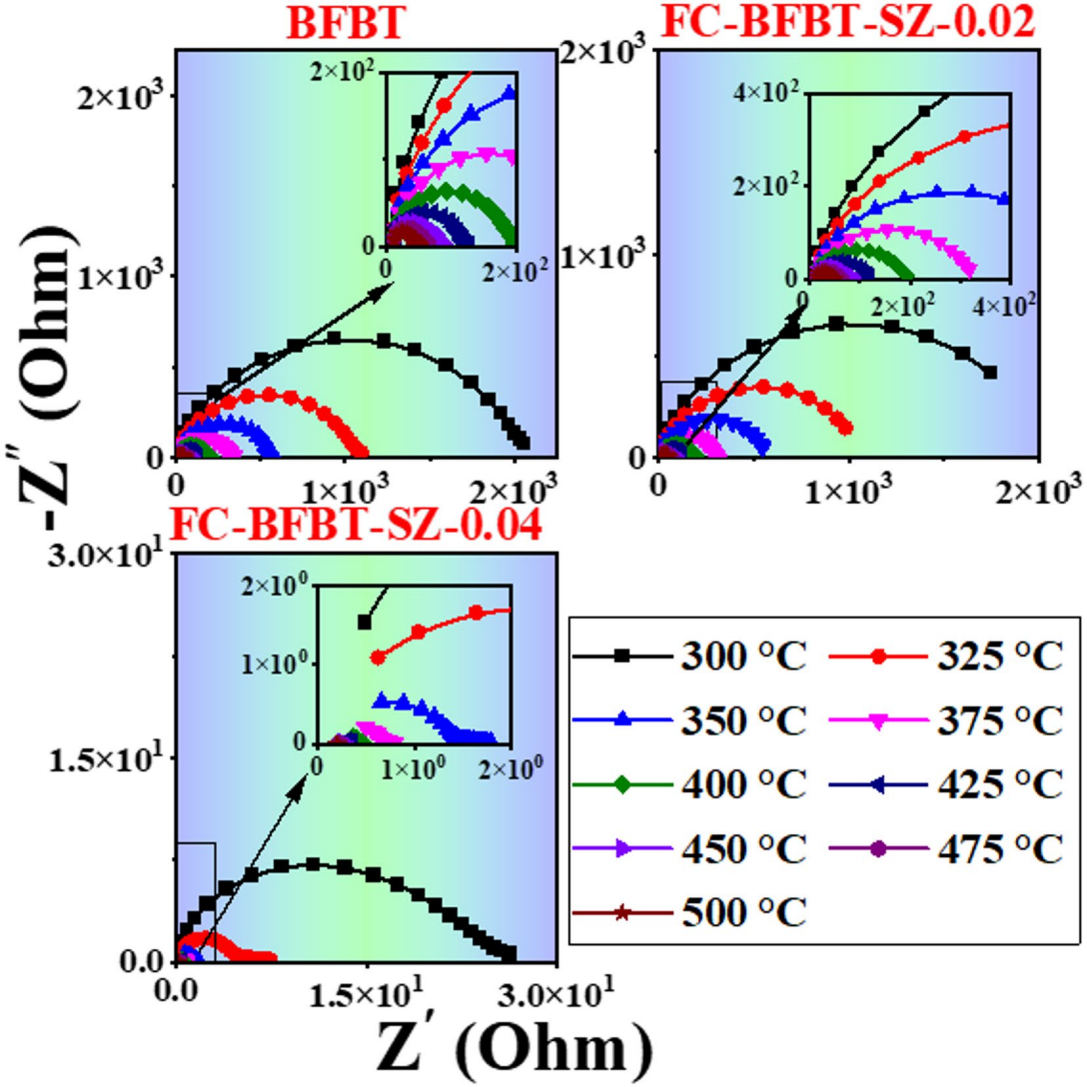
Nyquist (Cole–Cole) plots of furnace-cooled (FC–BFBT–SZ*x* ceramics *x* = 0, 02, and 04 mol%) recorded at different temperatures. The undoped sample shows a single semicircle corresponding to grain (bulk) conduction, while SZ-doped samples exhibit two arcs, indicating both grain and grain boundary contributions. A decrease in arc radius with increasing temperature suggests enhanced conductivity due to thermally activated charge carriers and oxygen vacancy mobility.

The Nyquist or Cole–Cole plots for furnace-cooled (FC–BFBT–SZ*x* ceramics *x* = 0, 0.02, and 0.04 mol%) are shown in Fig. 9. The undoped BFBT sample exhibits a single well-defined semicircular arc across the investigated temperature range, indicating that electrical conduction is primarily governed by the grain (bulk) response of the ceramic^57^. In contrast, the SZ-doped samples (FC–BFBT–SZ–0.02 and FC–BFBT–SZ–0.04) display two distinct semicircular arcs in their impedance spectra. The high-frequency arc corresponds to the grain (bulk) contribution, while the low-frequency arc arises from grain boundary effects. The resistance values associated with grains (Rg) and grain boundaries (Rgb) can be quantitatively determined by extrapolating the intercepts of the respective semicircles onto the real axis (Z′) of the Cole–Cole plot. The appearance of the second arc upon SZ doping suggests that the introduction of SrZrO_3_ leads to the development of additional resistive barriers at the grain boundaries, likely due to modifications in microstructure and defect chemistry^58,59^.

As the temperature increases, a consistent decrease in the radius of semicircular arcs is observed for all compositions. This reduction indicates enhanced electrical conductivity at elevated temperatures, attributed to increased charge carrier mobility. The improvement in conductivity with temperature is a result of thermally activated processes, particularly the movement of intrinsic charge carriers and oxygen vacancies^60^. Oxygen vacancies become more mobile at higher temperatures, contributing significantly to charge transport across grain boundaries^61^. These results confirm that SZ doping modulates the electrical microstructure of BFBT ceramics, introducing additional grain boundary resistance, while furnace cooling preserves distinct grain and grain boundary impedance responses. The dual-arc behavior in doped samples highlights the complex conduction mechanisms present in the system and underscores the role of compositional tuning in tailoring electro-ceramic performance^62–64^.

### Negative temperature coefficient of resistance (NTCR) behavior

**Fig. 10:**
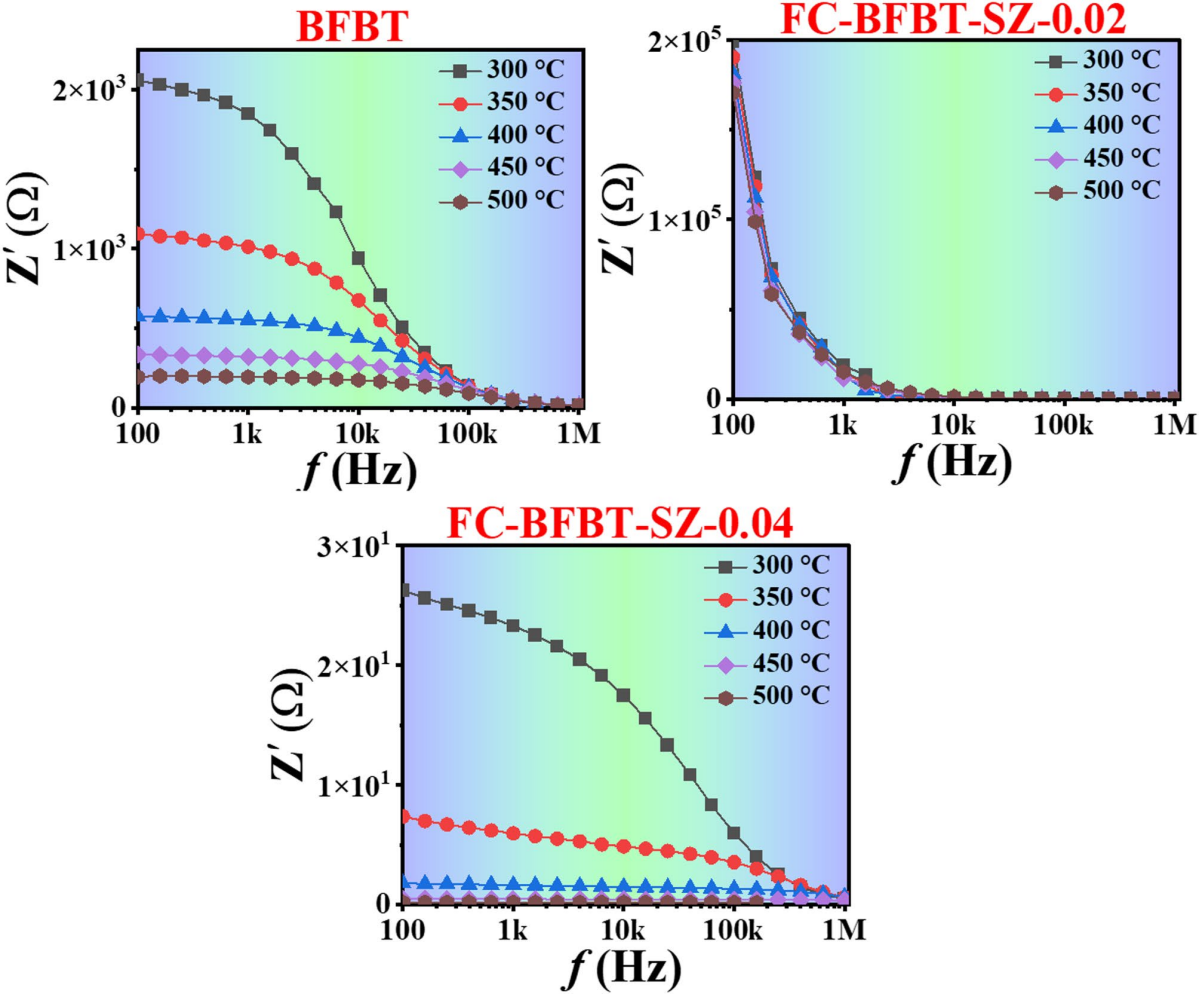
Frequency dependence of the real part of impedance (Z′) of furnace-cooled (FC–BFBT–SZ*x* ceramics *x* = 0, 0.02, and 0.04 mol%) at various temperatures (300–500 ℃), showing the transition from insulating to conductive behavior with increasing frequency.

The real component impedance (Z′) Bode plot shows the negative temperature coefficient of resistance (NTCR) behavior, which is the reduction in electrical resistance with temperature rise. Figure 10. illustrates the variation of the real part of impedance (Z′) as a function of frequency for the furnace-cooled (FC–BFBT–SZ*x* ceramics *x* = 0, 02, and 04 mol%) samples over a temperature range of 300–500 ℃. All samples exhibit a general trend where Z′ decreases with increasing frequency and temperature, indicating the thermally activated conduction mechanism of the material. At lower frequencies and temperatures, the impedance values are higher, implying the dominance of insulating behavior due to restricted charge carrier mobility. As the frequency increases, the impedance values drop significantly, reflecting the increased ease of charge carrier hopping and reduced resistance within the grain bulk. A notable observation across all compositions is that beyond approximately 400 kHz, the impedance curves for different temperatures converge and flatten. This flattening suggests that, at high frequencies, the grain response dominates, and the overall impedance becomes frequency- and temperature-independent, signifying enhanced conductivity. At lower frequencies, the impedance is mainly influenced by grain boundary resistance, whereas at higher frequencies, the contribution from the grain interior governs the electrical response. The observed decrease in Z′ with rising temperature further confirms that the furnace-cooled samples exhibit thermally activated conduction behavior, with increased charge carrier mobility at elevated temperatures^65^.

### High-temperature A.C. conductivity analysis

**Fig. 11:**
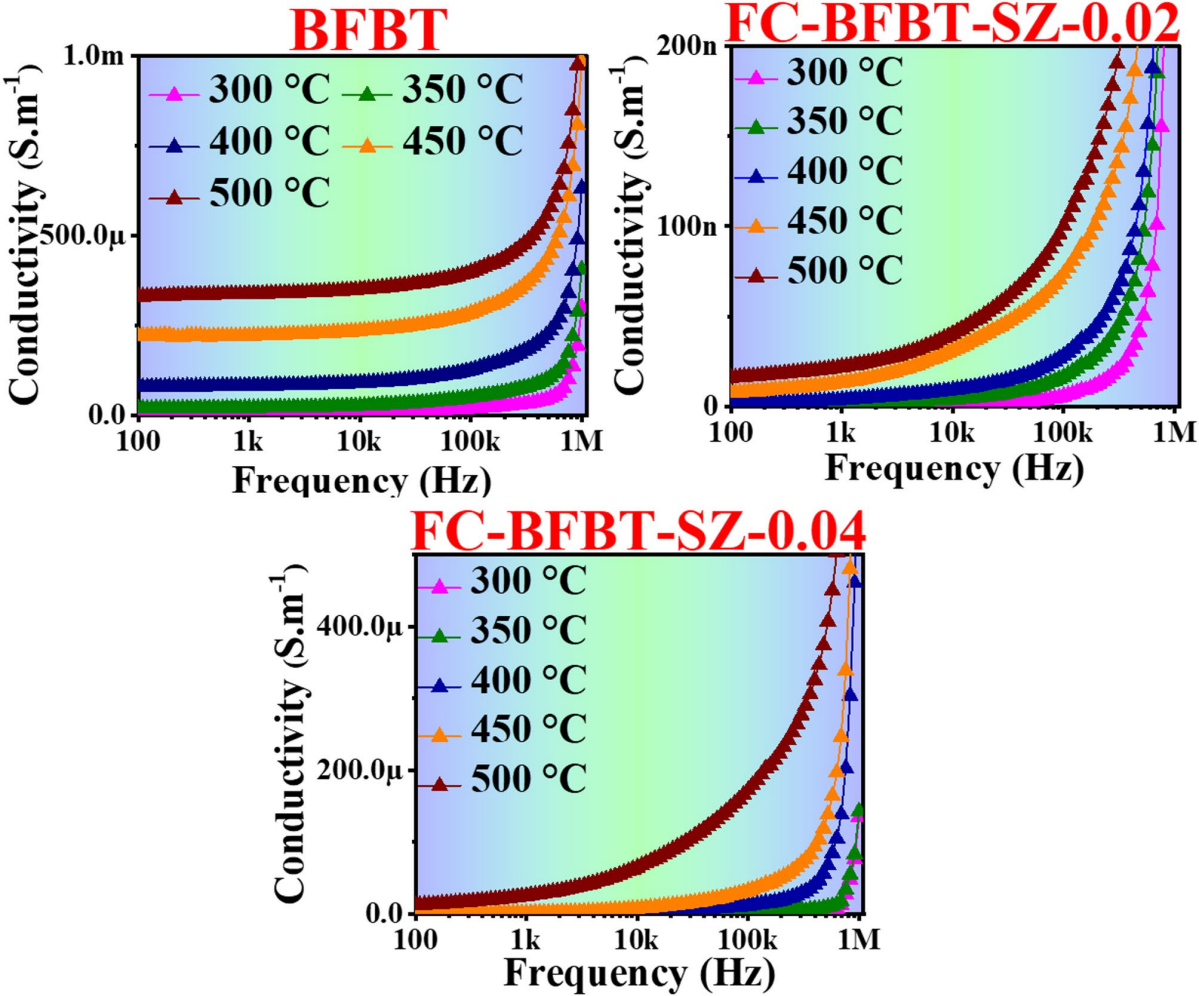
Frequency- and temperature-dependent conductivity for furnace-cooled FC–BFBT–SZ*x* ceramics with *x* = 0, 0.02, and 0.04 mol%. The results demonstrate enhanced conductivity with increasing SZ content and thermal activation, indicating improved charge transport through doping and elevated temperature.

The conductivity behavior of furnace-cooled BFBT ceramics and their SZ-doped variants (FC–BFBT–SZ–0.02 and FC–BFBT–SZ–0.04) was studied as a function of frequency and temperature and is represented in Fig. 11. Conductivity measurements performed over the 300–500 °C range reveal the influence of thermal energy on charge transport mechanisms. For the undoped BFBT sample, conductivity increases with both frequency and temperature, though it remains lower than that of the SZ-doped samples. This indicates that intrinsic charge carrier mobility in BFBT is limited and that conduction is primarily governed by thermally activated processes^29,66^. In all samples, conductivity improves with increasing frequency, attributed to the enhanced release of trapped charge carriers and more efficient hopping conduction^67^. SZ doping significantly enhances the electrical conductivity, particularly in FC–BFBT–SZ–0.02, where reduced grain boundary resistance and improved carrier mobility contribute to a noticeable increase compared to undoped BFBT^68^. At elevated temperatures, especially at 450 °C and 500 °C, the conductivity enhancement becomes more pronounced. The FC–BFBT–SZ–0.04 composition exhibits the highest conductivity among the samples, attributed to its higher SZ content. This promotes the formation of oxygen vacancies, thereby reducing defect-related losses and facilitating ionic transport^66,69^. The observed increase in conductivity with temperature and frequency is consistent with a thermally activated hopping mechanism, where thermal energy promotes charge carrier excitation and movement through the lattice^67^. Furthermore, the frequency-dependent conductivity response of all samples was examined using Jonscher’s Universal Power Law,

$${\sigma}\left({\omega}\right)\text{}\text{=}\text{}{{\sigma}}_{\text{dc}}\text{}\text{+}\text{}\text{A}{{\omega}}^{\text{n}}$$

and the plots were found to obey this relation well. A frequency-independent plateau corresponding to *σ*_dc_ was observed at low frequencies, followed by a dispersive region at higher frequencies. The power law exponent *n*(0 < *n* < 1) decreased with increasing temperature, which is characteristic of thermally activated hopping conduction and supports the correlated barrier hopping (CBH) model. Compared to undoped BFBT, the SZ-doped samples exhibited slightly lower nnn values, suggesting enhanced hopping transport due to increased oxygen vacancies and reduced grain-boundary barriers^70,71^. Overall, these findings confirm that SZ doping enhances the conductivity of furnace-cooled BFBT ceramics by improving carrier mobility and reducing grain boundary impedance. Among the studied compositions, FC–BFBT–SZ–0.04 shows the most promising potential for high-temperature conductive applications, making it suitable for use in electronic and dielectric devices^29,68^.

### Activation energy

**Fig. 12:**
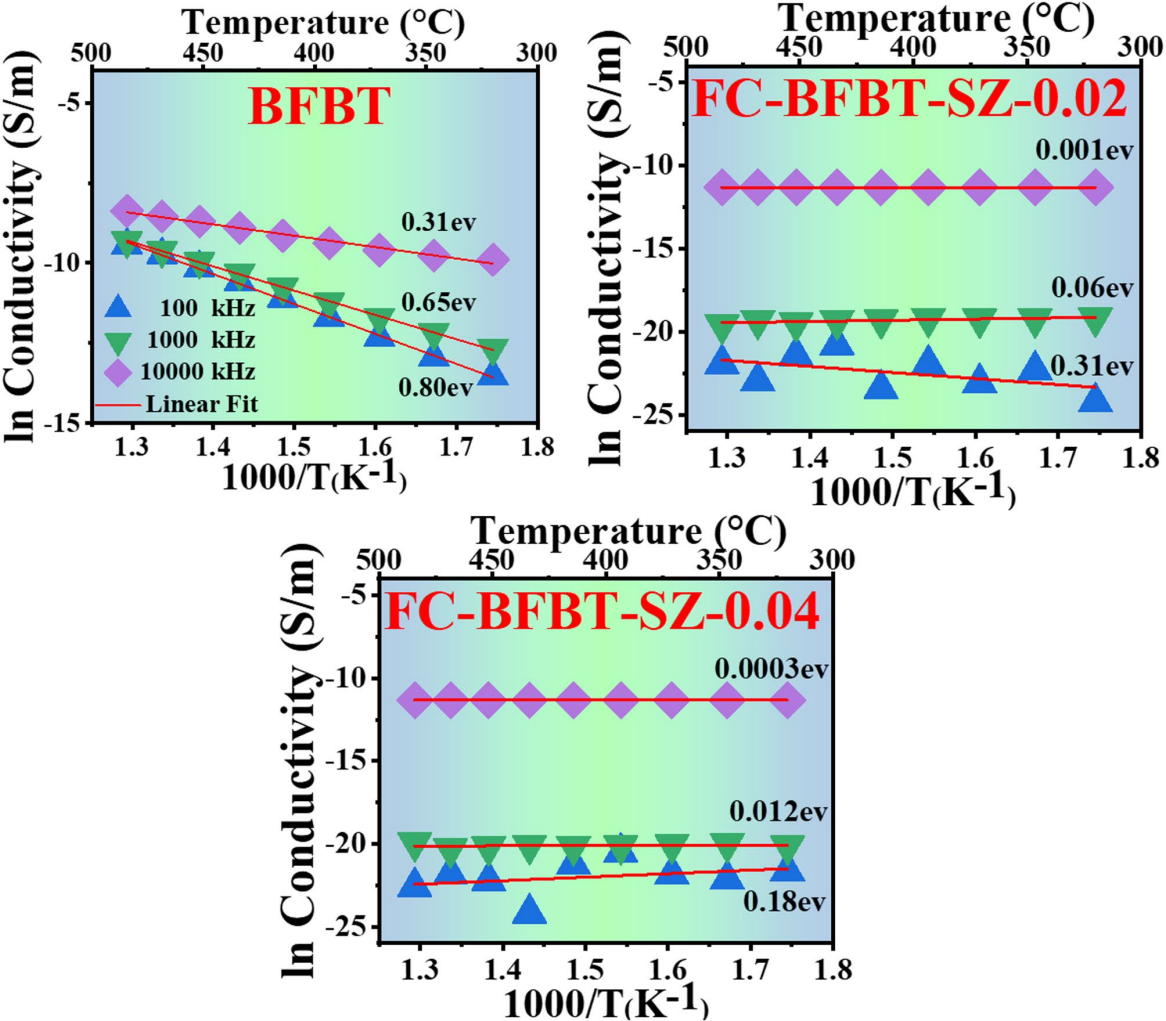
Activation energy variation of for furnace-cooled FC–BFBT–SZ*x* ceramics with *x* = 0, 0.02, and 0.04 mol%, showing the reduction in activation energy with increasing SZ content, indicating improved conduction behavior.

The activation energy analysis of furnace-cooled BFBT composites, as illustrated in Fig. 12, highlights the impact of SZ dopant concentration on the conduction mechanism as a function of temperature and frequency. A systematic reduction in activation energy is observed with increasing SZ content from 0 to 0.02 mol% and 0.04 mol%, suggesting enhanced electrical conductivity and reduced thermal resistance^72,73^. In the undoped BFBT (FC–BFBT–SZ–0) sample, conduction is predominantly thermally activated, indicating that charge carriers—either ions or electrons—require considerable thermal energy to overcome inherent energy barriers. This behavior is associated with a low free carrier concentration and the presence of microstructural impediments such as grain boundaries, which restrict charge transport^68^. Doping with 0.02 mol% (FC–BFBT–SZ–0.02) alters the conduction mechanism by inducing microstructural modifications. The incorporation of SZ promotes the formation of oxygen vacancies and partially mitigates grain boundary resistance. These changes create favorable conditions for localized charge carrier motion, especially at higher frequencies where the alternating electric field facilitates hopping conduction. As a result, the activation energy required for carrier transport is significantly reduced^29,67^. Further doping to 0.04 mol% SZ (FC–BFBT–SZ–0.04) intensifies this effect. The material exhibits a more optimized microstructure with a higher density of conduction pathways and beneficial defect sites. At this level, charge transport becomes increasingly reliant on defect-mediated mechanisms such as polaron hopping and tunneling, particularly at high frequencies. The pronounced decrease in activation energy from ~ 0.80 eV in undoped BFBT to ~ 0.20 eV in SZ-doped variants suggests a fundamental shift in the conduction mechanism. In the undoped system, conduction is dominated by thermally activated ionic transport across grain boundaries, which requires higher thermal energy. With SZ incorporation, the substitution of Zr^4+^ promotes oxygen vacancy formation and stabilizes mixed-valence Fe^3+/^Fe^2+^ states. These defects provide localized hopping sites and enable small-polaron hopping conduction, both of which demand significantly lower thermal energy. Consequently, the reduction in Ea reflects a transition from grain-boundary-limited ionic conduction to vacancy- and polaron-mediated hopping conduction^68,74^. he observed trend indicates a transition in the dominant conduction mechanism—from purely thermally activated behavior in undoped BFBT to a mixed conduction regime in SZ-doped variants, governed by both defect states and frequency response. This shift contributes to enhanced conductivity and thermal stability, making SZ-doped BFBT ceramics promising materials for high-temperature electronic applications requiring consistent electrical performance across broad thermal ranges^29,74^.

## Conclusion

In this study, SrZrO_3_-modified BiFeO_3_–BaTiO_3_ ceramics were successfully synthesized through a conventional solid-state route followed by furnace cooling. The introduction of Sr^2+^ and Zr^4+^ ions was found to refine grains, enhance densification, and suppress abnormal grain growth. XRD, EDS, and FTIR collectively confirmed the formation of a single-phase perovskite structure with reliable elemental incorporation. Dielectric measurements showed that, although the maximum permittivity decreased with doping, the materials exhibited improved thermal stability, reduced dielectric loss, and more uniform relaxation behavior. Impedance analysis further revealed the distinct contribution of grains and grain boundaries, while conductivity studies confirmed adherence to Jonscher’s Universal Power Law with reduced activation energy. Overall, the 4 mol% SrZrO_3_ composition demonstrated the best balance of structural integrity, dielectric efficiency, and conduction stability, highlighting the potential of furnace-cooled BFBT–SZ ceramics for reliable high-temperature capacitor and electronic applications.

## Data Availability

The datasets used and/or analysed during the current study available from the corresponding author on reasonable request.
